# Development of a Fully Automated, High‐Throughput Molecular Assay for Detection of Rat Hepatitis E Virus in Routine Diagnostics

**DOI:** 10.1002/jmv.70824

**Published:** 2026-02-09

**Authors:** Jessica Panajotov, Katja Giersch, Lisa Sophie Pflüger, Dominik Nörz, Moritz Grunwald, Hui Ting Tang, Marco Kaiser, Sven Pischke, Rainer G. Ulrich, Susanne Pfefferle, Julian Schulze zur Wisch, Victor Max Corman, Martin Aepfelbacher, Reimar Johne, Marc Lütgehetmann

**Affiliations:** ^1^ German Federal Institute for Risk Assessment (BfR) Berlin Germany; ^2^ Institute of Medical Microbiology, Virology and Hygiene University Medical Center Hamburg‐Eppendorf (UKE) Hamburg Germany; ^3^ TIB‐Molbiol Syntheselabor GmbH Berlin; ^4^ Department of Medicine University Medical Center Hamburg‐Eppendorf (UKE) Hamburg Germany; ^5^ German Center for Infection Research (DZIF) Hamburg‐Lübeck‐Borstel‐Riems site Germany; ^6^ Friedrich‐Loeffler‐Institut (FLI), Institute of Novel and Emerging Infectious Diseases Greifswald‐Insel Riems Germany; ^7^ Institute of Virology Charité‐Universitätsmedizin Berlin, corporate member of Freie Universität Berlin and Humboldt‐Universität zu Berlin Berlin Germany; ^8^ German Centre for Infection Research (DZIF), associated partner site Charité Berlin Germany; ^9^ Labor Berlin‐Charité Vivantes Berlin Germany

**Keywords:** IVDR, ratHEV, *Rocahepevirus ratti*, RT‐qPCR

## Abstract

Recently, cases of human infection with rat hepatitis E virus (ratHEV, *Rocahepevirus ratti*) have been reported worldwide. Due to the significant genetic differences between ratHEV and human HEV genotypes 1–4 (*Paslahepevirus balayani*), current HEV diagnostic assays are unable to detect ratHEV. The aim was to establish and validate a laboratory‐developed ratHEV RT‐qPCR assay for use with human plasma and stool samples on a fully automated, high‐throughput platform. Published primers and probes were optimized for use on cobas 5800/6800/8800 systems using European Union In Vitro Diagnostics Regulation (IVDR)‐grade reagents, including an RNA full‐process inhibition control. Analytical sensitivity (21 repeats), linear range (five repeats) and precision (three repeats over 3 days) were evaluated using viral particles from cell culture (MN450851.1). The inclusivity was verified using DNA oligonucleotides and known positive samples (rat liver and human serum). The limits of detection were 98.9 copies/ml in plasma and 60.3 copies/ml in stool, and the assay showed excellent linearity over at least 5 log (r^2^: 0.991 in plasma and 0.9989 in stool) and high precision (< 0.62 ct). The assay reliably detected different ratHEV C1 subgenotypes, returning positive results for all 11 rat liver samples and one known ratHEV RNA‐positive human plasma sample, while no false positives were detected in the broad cross‐reactivity set (*n* = 41). In the pilot ratHEV surveillance cohort, 1.1% of plasma samples (*n* = 1999) were positive for HEV RNA, but none were positive for ratHEV RNA. Our new, fully automated, lab‐developed ratHEV assay can be used in compliance with the IVDR for routine human diagnostics. Further studies are needed to determine the clinical relevance in different human cohorts.

**Abbreviations**
ANOVA: analysis of variance
BfR: German Federal Institute for Risk Assessment
CE‐IVD: conformité Européene‐in vitro diagnostic devices
ct: threshold cycle
CV: coefficient of variation
dcp: digital copies
DNA: deoxyribonucleic acid
EBV: Epstein‐Barr virus
EDTA: ethylenediaminetetraacetic acid
EU IVDR: European Union In Vitro Diagnostics Regulation
FLI: friedrich‐loeffler‐institut
GE: genome equivalents
HSV: herpes simplex virus
Ig: immunoglobuline
IU: international units
LoD: limit of detection
MMX‐R: master mix reagent
NAT: nucleic acid test
pt: patient
ratHEV: rat hepatitis E virus
RNA: ribonucleic acid
RT‐qPCR: reverse transcription quantitative polymerase chain reaction
SD: standard deviation
UNG: uracil‐N glycosylase
VZV: varicella zoster virus
WHO: World Health Organization

## 1 Introduction

Viruses of the subfamily *Orthohepevirinae*, such as hepatitis E virus (HEV, species *Paslahepevirus balayani*), infect humans and different animals worldwide, and cause acute and chronic hepatitis as well as a broad spectrum of extrahepatic manifestations [1, 2].

In 2010, a novel hepevirus, which is currently classified as *Rocahepevirus ratti* was found in wild Norway rats (*Rattus norvegicus*) from Germany [3, 4]. *Rocahepevirus ratti* genotype C1 (hereafter used synonymously with ratHEV) has also been detected in other rodents (*Rattus* sp., *Bandicota indica), eulipotyphlids* (Asian house or musk shrew, *Suncus murinus*), while genotype C2 has been found in mustelids (ferret and mink) [5]. A so far not further classified genotype C3 has been found in Chevrier's field mouse (*Apodemus chevrieri*) [6] and genotype C4, classified in species *Rocahepevirus eothenomi*, has been described in voles [7, 8]. Orthohepeviruses of other genera also occur in bats (*Chirohepevirus)* or birds (*Avihepevirus*). Interestingly, in the last years, ratHEV RNA was detected in humans from Hong Kong, Canada, Spain, Germany and France and was associated with clinical symptoms similar to those caused by HEV [9, 10, 11, 12, 13, 14, 15, 16, 17, 18, 19, 20]. RatHEV is now recognized an emerging zoonotic pathogen with around 30 known global human cases.

Although *Rocahepevirus ratti* genotype C1 is phylogenetically distinct from the *Paslahepevirus* genus with an average similarity of the nucleotide sequence of only 55‐60% [21, 22], a recent study shows that the molecular basis for the tropism in human target cells is an unexpected shared partial antigenic overlap of both viruses on the capsid protein [20]. Interestingly, this sequence similarity is absent in the closely related *Rocahepevirus genotype* C2 (ferret, mink), and batHEV and avian HEV, members of other genera of the *Orthohepevirinae* subfamily show little or no binding to human cells in in vitro assays [23, 24].

Due to the genomic differences between *Paslahepevirus balayani* and *Rocahepevirus ratti*, established RT‐qPCR assays for HEV RNA are likely to miss ratHEV detection [25]. Moreover, a recent study demonstrated that published in‐house ratHEV RT‐qPCR assays vary strongly in their ability to detect ratHEV RNA positive samples (ranging from 6.0% to 72.0%) [12]. Therefore, there is a gap in diagnostic tools that need to be validated for routine use in human samples.

To enable detection of ratHEV RNA in clinical samples and to better asses the clinical relevance of human ratHEV infections, we established and validated a lab‐developed RT‐qPCR assay in accordance with the European Union In Vitro Diagnostics Regulation (2017/746 EU IVDR) for the use on the high throughput, fully automated cobas 5800/6800/8800 system (Roche, Mannheim, Germany).

## 2 Materials Methods

### 2.1 Design and Setup of In‐House RatHEV RT‐qPCR Assay

A primer/probe set, detecting different ratHEV subgenotypes and published by Sridhar et al. [16], was selected and modified for use on cobas 5800/6800/8800 systems: HEC‐FO1: 5'‐CCG ATG GAG ACA CAT CAR TAT (Ometh‐G)T, HEC‐qual‐FO2: 5'‐CTT GTT GAG CTY TTC TCC C(Ometh‐C)T, HEC‐qual‐RO: 5'‐TGT ACC GGA TGC GAC (Ometh‐C)AA and HEC‐probe: 5'‐FAM‐TGC AGC TTG (ZEN) TCT TTG ARC CCG C‐IBFQ. To reduce the risk of primer dimer formation, the primers were modified with 2′‐O‐methyl RNA bases within the 3′ region. Primers were custom‐made by Ella Biotech (Fuerstenfeldbruck, Germany). Adding two GC nucleotides to the probe sequence increased its melting temperature by > 6°C, thereby enhancing its robustness against mismatches. The cobas software uses a unique automated result calling approach by validating the shape of the curve (s‐form) and by normalizing the fluorescence signal of the probe against the background in order to calculate the increase in relative fluorescence intensity (RFI). The HEC‐probe is a double‐quenched probe with an internal ZEN quencher and Iowa Black FQ (IBFQ) at the 3’ end and was obtained from Integrated DNA Technologies (IDT) (Coralville, IA, USA). The additional internal ZEN quencher reduces baseline fluorescence, positively affecting the RFI ratio achievable with the assay. This concept of double‐quenched probes was also applied in our recent assays using the cobas5800/6800/8800 system [26, 27]. Relevant sequences were collected from GenBank, aligned and compared to the primer/probe sequences using Geneious 9.0 (Dotmatics, Boston, MA, USA, data of the alignment 11/2023).

The target primer and probe were added to the cobas omni utility channel reagent kit (Roche), which also contains the master mix reagents MMX‐R1 and MMX‐R2, an internal RNA full‐process control (IC), a protease, a polymerase with reverse transcriptase (RT) activity and an elution buffer. The IC is added automatically during sample processing to monitor extraction and amplification. Primers and probes for the IC are included in MMX‐R2. The kits were prepared according to the manufacturer's instructions and as described in supporting methods and Supporting Table 1a). The internal control is detected exclusively in channel 5. Absence of an IC fluorescence signal indicates possible inhibition and renders the PCR and target result invalid, even if a target signal was detected.

The following run protocol was used on cobas 5800/6800/8800: Uracil‐N gGlycosylase (UNG) incubation (predefined), pre‐PCR/RT step (1 cycle: 55°C 120 s, 60°C 360 s, 65°C 240 s), 1st measurement (5 cycles: 95°C 5 s, 55°C 30 s), 2nd measurement (45 cycles: 91°C 5 s, 58°C 25 s), cooling (predefined). The relative fluorescence intensity (RFI) for ratHEV on channel 2 was 1.5 (cut‐off) and for the internal control on channel 5 2.0 (cut‐off). The measured sample volume was 850 µl. The cut‐off was determined by comparing the RFI of negative samples with the RFI of positive controls, such as gene blocks (*see also below*) and should be at least three times higher than RFIs obtained from negative controls.

### 2.2 Evaluation of Analytical Performance

To determine the analytical sensitivity, linearity and precision of the new assay a standard was generated by spiking a cell lysate from Huh7‐Lunet‐BLR cells persistently infected with an infectious clone based on the TIB‐MOLBIOL Syntheselabor GmbH is Part of Roche Group. sequence of ratHEV‐strain pt2 (accession number: MN450851.1) [28], originally identified in a human patient in Hong Kong [15] and belonging to *Rocahepevirus ratti* subgenotype C1b, in EDTA‐plasma and stool matrices. Cell lysate was generated by three times freeze at −80°C for at least 30 min and thaw, and was solved in phosphate‐buffered saline (PBS, Pan Biotech GmbH, Aidenbach, Germany). Nucleic acids from standard were extracted on a MagNA‐Pure 96 instrument (Roche Diagnostics) and quantified by digital‐PCR using the Qiacuity (Qiagen, Hilden, Germany) digital‐PCR system according to manufacturer's instructions and the primers and probe that was used for the cobas assay (*see above*). The unit of the standards is digital copies/ml (dcp/ml). EDTA‐plasma samples were measured undiluted, while stool samples were treated prior to analysis as previously described [27]. Briefly, a swab was directly dipped into the stool sample, transferred to one tube of cobas PCR medium (4.2 ml) (Roche Diagnostics, Rotkreutz, Switzerland) and vortexed (direct swab stool sample preparation method).

Analytical sensitivity in EDTA‐plasma and stool was determined by serial two‐fold dilution of the cell cultured‐derived ratHEV‐pt2 virus standard using eight dilution steps and 21 repeats (plasma) and eight repeats (stool) per dilution. Linearity was assessed by ten‐fold serial dilution. Linearity in plasma and stool was evaluated using ratHEV‐pt2 standard and ten‐fold serial dilutions (at least five dilution steps, *n* = 5 per dilution).

Intra‐ and inter‐run variability was assessed using ratHEV‐pt2 plasma standard in different concentrations: two samples with high (21.8ct and 25.2ct) and three samples with medium/low (28.8ct, 32.4ct and 35.9ct) viral RNA concentration as well as one negative sample. Each sample was tested in triplicates in three runs over 3 days. Within‐lab precision was calculated as sum of squares of precision components. Precision was calculated as standard deviation (SD) with coefficient of variation (CV%) according to ANOVA statistics using Validation Manager (Finbiosoft, version 2023.11.17).

Exclusivity was determined by measuring the WHO HEV RNA genotype panel for NAT‐based assay (#8578/13) consisting of 11 different HEV (sub)genotypes in plasma or stool (100 µl of each WHO standard were mixed with 1.5 ml cobas PCR medium (Roche)). A cross‐reactivity study was performed using 26 isolates of different common enteric bacteria (e.g. *Escherichia coli*, Enterococcus *faecium, Enterococcus gallinarum*) and 15 clinical samples containing viruses (e.g. Epstein‐Barr virus, EBV, herpes simplex virus type 1/2, HSV‐1/2, varicella zoster virus, VZV).

The inclusivity of the ratHEV assay was determined by measuring gBlocks or ultramer DNA oligonucleotides of different ratHEV subtypes and subgenotypes (*Rocahepevirus ratti* C1a, C1c, C1d and C2, *Rocahepevirus* sp. C3, *Rocahepevirus eothenomi* C4, Table 1) and cell lysates containing ratHEV‐pt2 C1a strain (accession number: MN450851.1) or ratHEV‐R63 (accession number: GU345042) C1a strain originally identified in a rat [21, 28]. In addition, 11 rat liver tissue samples and one human plasma sample pre‐tested positive for ratHEV RNA at the Federal Institute for Risk Assessment (BfR) (Berlin, Germany) and the Charité Berlin, respectively, were added to the inclusivity set [19, 29].

**Table 1:** Alignments of primers and probe of the ratHEV RT‐PCR assay against currently available Rocahepevirus ratti (genotypes/subgenotypes C1a‐C1d and C2) and other Rocahepevirus (genotype C3 and C4) sequences (*n* = 39).

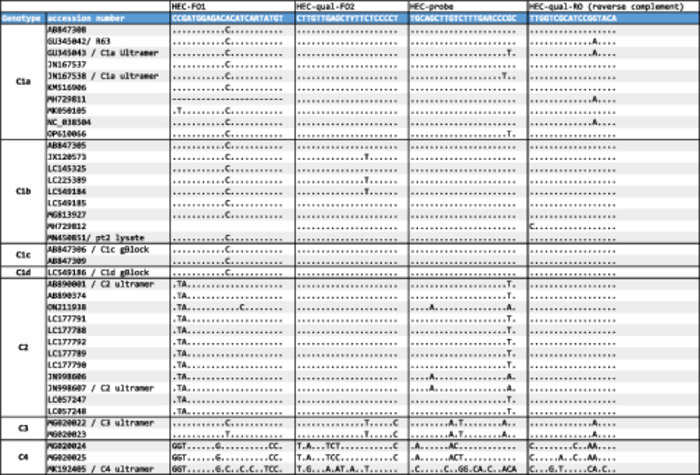

Prior measurement, 1 µl of gBlocks (IDT, Coralville, IA, USA) were spiked in 5 ml cobas PCR medium (Roche Diagnostics) supplemented with 200 µl carrier RNA (Qiagen). 4nmol ultramer DNA oligonucleotides (IDT, Coralville, IA, USA) were dissolved in 1.5 ml Nucleic Acid Dilution Buffer (Qiagen) and 1 µl was spiked in 1.5 ml PCR medium (Roche Diagnostics). One hundred µl cell lysate was mixed with 1.5 ml cobas PCR medium (Roche Diagnostics). Rat liver tissue samples were lysed (Precellys 24, Bertin, Rockville, MD, USA) using 2 ml tubes prefilled with ceramic beads (Precellys Lysing Kit) and mixed with 4.5 ml cobas PCR medium (Roche Diagnostics). The human plasma sample was diluted in PBS (1:40) and cobas PCR medium (Roche Diagnostics) (1:8).

### 2.3 Routine Diagnostic HEV RT‐qPCR Assays

To verify that diagnostic HEV (*Paslahepevirus balayani*) assays do not detect ratHEV (*Rocahepevirus ratti*) highly positive gBlocks or ultramer DNA oligonucleotides containing different *Rocahepevirus* genotypes, the two cell lysates ratHEV‐pt2 and ratHEV‐R63, two infected rat liver tissue samples and one human plasma sample were measured using a CE‐IVD HEV assay (HEV cobas test, Roche diagnostics) and our in‐house HEV assay on cobas 6800/8800 systems. The CE‐IVD Roche HEV cobas test is certified to detect HEV RNA genotypes 1‐4 in plasma samples from blood donors and was used in accordance with the manufacturer's recommendations. The measured sample volume was 850 µl and the limit of detection (LoD) was 18.6 interntional units (IU)/ml.

Our in‐house pan HEV RT‐qPCR assay is based on primer and probes from *Garson* et al. detecting the HEV open reading frame (ORF) 2/3 region (HEV_FO: 5'‐GGT GGT TTC TGG GGT GA(OMe‐C), HEV_R: 5'‐AGG GGT TGG TTG GAT GAA, HEV‐probe: 5'‐HEX‐TGA TTC TCA GCC CTT CGC‐MGB‐BMNQ‐535) and are validated for clinical plasma and stool samples on cobas 5800/6800/8800 systems [30]. The manufacturing protocol can be found in Supporting Table 1b. Run protocol was identical to the protocol used for ratHEV (see above). The RFI for HEV on channel 3 was 2.0 and for the internal control on channel 5 2.5. The measured sample volume was 850 µl and the LoD was 6.0 IU/ml. The linear range was determined with the WHO HEV standard and was 24 IU/ml – 1 × 10^8^ IU/ml.

### 2.4 Pilot RatHEV Surveillance

A total of 1999 anonymized remnant clinical plasma samples from 1773 individuals with a physician's request for HEV RNA measurement were measured using the ratHEV assay. Serum anti‐HEV IgG and IgM antibodies were measured using the LIAISON® murex anti‐HEV IgG and anti‐HEV IgM assays (DiaSorin Deutschland GmbH, Dietzenbach, Germany) on the LIASON XL platform according to the manufacturer's instructions. The use of patient material was conducted in accordance with §12 of the Hamburg hospital law (§12 HmbKHG). The use of anonymized remnant diagnostic samples from patients was approved and informed consent was waived by the ethics committee of the Hamburg Medical Association (PV5626).

## 3 Results

### 3.1 Alignments of Primer/Probe Against Current Target Sequences

Primer/probe sequences were analysed for mismatches among currently available *Rocahepevirus ratti* (genotypes C1 and C2) and other *Rocahepevirus* species (genotype C3 and C4) sequences (*n* = 39). Due to the heterogeneity of zoonotic ratHEV genotype C1 sequences, a further subclassification into subgenotypes C1a‐d was applied [8].

C1 sequences showed no mismatch (C1c and C1d) or up to one mismatch per oligonucleotide (C1a, C1b) when aligned to the oligonucleotides of the ratHEV assay, indicating full coverage of the currently known ratHEV C1 sequences. *Rocahepevirus ratti* C2 sequences from ferrets (AB890374.1) demonstrated one (69%) or two mismatches (31%) in the probe and no mismatches in the primers, while other *Rocahepevirus* species genotype C3 and C4 sequences from Chevrier's field mouse and voles were not expected to be detected by the primers/probe (more than 5 mismatches per PCR product) (Table 1).

### 3.2 Analytical Sensitivity

**Figure 1:**
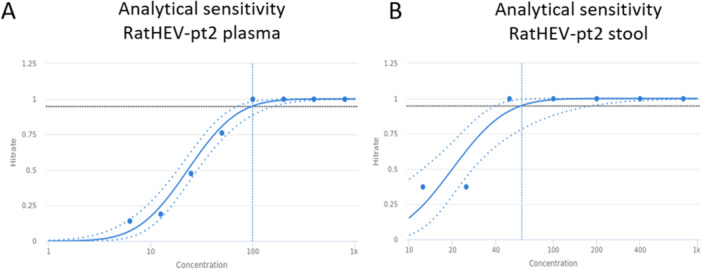
LoD (95% probit analysis) obtained from a twofold dilution of ratHEV‐pt2 containing cell culture lysates spiked in plasma (*n* = 21/dilution) (A) or stool matrix (B) (*n* = 8/dilution). Dashed black line: 0.95 hitrate. Blue line: probit curve. Dotted blue lines: Confidence intervals. Blue dots: hitrates.

Cell culture lysates containing ratHEV subtype C1b strain pt2 were used for evaluation of the analytical performance. 5% Probit analysis (CLSI EP17‐A2) was used to determine the LoD, which was 98.9 dcp/ml (confidence interval: 71.3‐162.0 dcp/ml) in EDTA‐plasma (Figures 1A) and 60.3 dcp/ml (confidence interval: 39.8‐188.0 dcp/ml) in stool (Figure 1B). Of note, although the LOD of stool is slightly lower than plasma the confidence intervals of both matrices are overlapping.

### 3.3 Linearity

**Figure 2:**
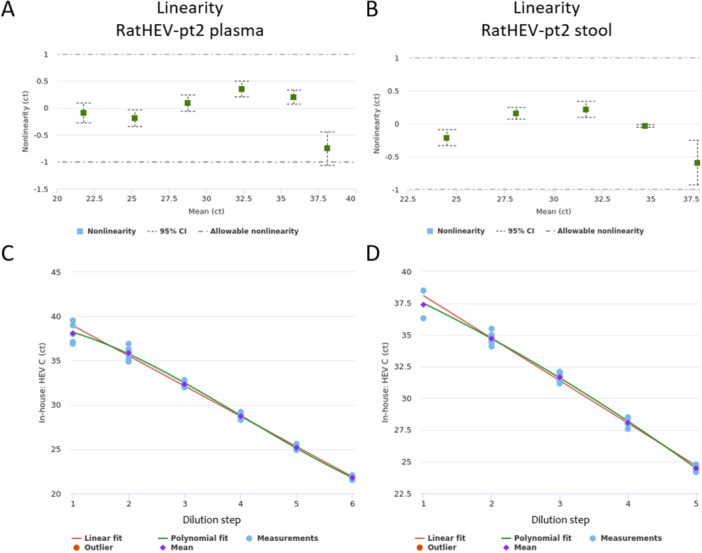
Linearity dot blot diagram of ratHEV‐pt2 RNA detection in spiked plasma (A) and stool (B). The allowable non‐linearity range is shown as dotted line (± 1 ct). The graphs show the best fit (polynomial fit) for all dilution steps according to the mean value in plasma (C) and stool (D).

Linearity was assessed in EDTA‐plasma and stool over at least five log‐steps through comparing linear fit to higher order polynomial fit mean ct values for each individual dilution step. Linear range was 21.8ct − 38.1ct (3rd polynomial order) in plasma (Figure 2A,C) and 24.5ct − 37.4 ct (2nd polynomial order) in stool matrix (Figure 2B,D). Linear regression slopes and r^2^ were −3.41 and 0.991 in plasma and −3.35 and 0.9989 in stool matrix, respectively.

### 3.4 Precision

For analytical precision triplicates of highly positive (*n* = 2), medium/low positive (*n* = 3) and negative samples (*n* = 1) were measured over three consecutive days and analysed with Validation Manager (Finbiosoft, version 2023.11.17). The intra‐ (within‐run) and inter‐run (between‐day) variability was below 0.25ct at the high concentration and below 0.62ct at the low concentration. The negative sample remained negative on all 3 days (Table 2).

**Table 2:** The intra‐ (within‐run) and inter‐run (between‐day) precision (ANOVA) of the in‐house ratHEV RT‐qPCR assay. For each sample triplicates were measured on three different days. CV, coefficient of variation (in %); SD, standard deviation (in ct).

Sample	Unit	*N*	Mean	Within‐Run		Between‐Day	
				SD	%CV	SD	%CV
HEC_high1	ct	9	21.8	0.180 (0.116–0.395)	0.825% (0.531%–1.82%)	0.131 (0.054–15.8)	0.6% (0.248%–72.6%)
HEC_high2	ct	9	25.2	0.160 (0.103–0.352)	0.634% (0.408%–1.4%)	0.161 (0.0736–3.69)	0.638% (0.292%–14.6%)
HEC_low1	ct	9	32.4	0.141 (0.0911–0.311)	0.437% (0.282%–0.962%)	0.252 (0.125–2.26)	0.778% (0.387%–6.99%)
HEC_low2	ct	9	35.9	0.561 (0.361–1.230)	1.56% (1.01%–3.44%)	0.254 (N/A)	0.708% (N/A)
HEC_medium	ct	9	28.8	0.153 (0.0984–0.336)	0.531% (0.342%–1.17%)	0.274 (0.136–2.45)	0.95% (0.474%–8.51%)
HEC_neg	ct	9	0	0	0%	0	0%

### 3.5 Inclusivity

To verify the detection of different *Rocahepevirus* genotypes, cell culture lysates, gBlocks or ultramer DNA oligonucleotides containing *Rocahepevirus* (sub)genotypes C1a, C1c, C1d, C2 (ferret), C3 (Chevrier's field mouse) and C4 (vole) were applied to our new assay. All *Rocahepevirus ratti* genotype C1 samples were measured positive by our ratHEV assay (ct 8.1−27.2) (Table 3). Genotype C2 with one mismatch in the probe (AB890001) was positive, while genotype C2 with two mismatches in the probe (JN998607) was positive with an RFI clearly above 1.5 (cut‐off), but called negative by the software due to a non‐sigmoidal curve, indicating suboptimal performance of the probe (Table 3). Other *Rocahepevirus* genotypes (C3 and C4), which are mainly found in field mice and voles and have not been detected in humans so far, were not detected by our assay (Table 3).

**Table 3:** Inclusivity and exclusivity set. Inclusivity: RatHEV positive controls (two cell culture lysates, two gBlocks and six ultramer DNA oligonucleotides containing different ratHEV genotype and subgenotype sequences) and ratHEV positive samples (two rat livers and one patient plasma). All controls and samples were measured using the new ratHEV assay, our HEV in‐house assay and a CE‐IVD HEV assay (Roche) on cobas 5800/6800/8800 systems. Exclusivity: HEV positive controls from the WHO HEV genotype panel with 11 samples. *was called negative by the cobas software due to non‐sigmoidal curve.

	Sample	Species	Genotype	ratHEV in‐house	HEV in‐house	HEV CE‐IVD Roche
Inclusivity set	raHEV‐R63 cell culture lysate (GU345042)	*Rocahepevirus ratti*	C1a	pos (ct 22.6)	neg	neg
	ratHEV‐pt2 cell culture lysate (MN450851.1)	*Rocahepevirus ratti*	C1b	pos (ct 27.2)	neg	neg
	gBlock (AB847306)	*Rocahepevirus ratti*	C1c	pos (ct 10.8)	neg	neg
	gBlock (LC549186)	*Rocahepevirus ratti*	C1d	pos (ct 10.6)	neg	neg
	ultramer (GU345043)	*Rocahepevirus ratti*	C1a	pos (ct 10.2)	neg	neg
	ultramer (JN167538)	*Rocahepevirus ratti*	C1a	pos (ct 8.1)	neg	neg
	ultramer (AB890001)	*Rocahepevirus ratti*	C2	pos (ct 9.4)	neg	neg
	ultramer (JN998607)	*Rocahepevirus ratti*	C2	pos*	neg	neg
	ultramer (MG020022)	*Rocahepevirus eothenomi*	C3	neg	neg	neg
	ultramer (MK192405)		C4	neg	neg	neg
	rat liver 1	*Rocahepevirus ratti*	C1	pos (ct 17.0)	neg	neg
	rat liver 2	*Rocahepevirus ratti*	C1	pos (ct 16.4)	neg	neg
	patient plasma	*Rocahepevirus ratti*	C1	pos (ct 31.9)	neg	neg
Exclusivity set	WHO HEV genotype panel 8567	*Paslahepevirus balayani*	1a	neg	neg	pos (ct 42.5)
	WHO HEV genotype panel 8568	*Paslahepevirus balayani*	1a	neg	pos (ct 33.3)	pos (ct 34.6)
	WHO HEV genotype panel 8569	*Paslahepevirus balayani*	1e	neg	pos (ct 37.9)	pos (ct 42.4)
	WHO HEV genotype panel 8570	*Paslahepevirus balayani*	3b	neg	pos (ct 33.6)	pos (ct 37.0)
	WHO HEV genotype panel 8571	*Paslahepevirus balayani*	3c	neg	pos (ct 37.0)	pos (ct 40.9)
	WHO HEV genotype panel 8572	*Paslahepevirus balayani*	3e	neg	pos (ct 35.6)	pos (ct 37.2)
	WHO HEV genotype panel 8573	*Paslahepevirus balayani*	3 f	neg	pos (ct 34.5)	pos (ct 36.4)
	WHO HEV genotype panel 8574	*Paslahepevirus balayani*	3 (rabbit‐like)	neg	pos (ct 29.6)	pos (ct 38.2)
	WHO HEV genotype panel 8575	*Paslahepevirus balayani*	4c	neg	pos (ct 34.8)	pos (ct 36.9)
	WHO HEV genotype panel 8576	*Paslahepevirus balayani*	4 g	neg	pos (ct 34.9)	pos (ct 38.4)
	WHO HEV genotype panel 8577	*Paslahepevirus balayani*	2a	neg	pos (ct 29.6)	pos (ct 34.4)

In addition, 11 rat liver tissue samples pre‐tested ratHEV RNA positive by the BfR were also positive using our RT‐qPCR assay [29]. The median ct value of ratHEV positive liver samples was 16.4 (range 11.7−30.1) (data not shown), indicating massive intrahepatic infection in rats. One human plasma sample pretested positive by the Charité Berlin was also ratHEV positive using our assay (ct 31.9) (Table 3) [19].

The ratHEV positive controls and samples (two representative high positive rat liver samples and the human plasma sample) were then applied to a CE‐IVD HEV assay and our in‐house HEV assay on cobas 5800/6800/8800 systems. HEV RNA could not be detected in any of these samples, confirming that diagnostic tests detecting HEV (*Paslahepevirus balayani)* are commonly not able to detect ratHEV (*Rocahepevirus ratti*) (Table 3).

### 3.6 Exclusivity and Cross‐Reactivity

Different human HEV genotypes from the WHO HEV RNA genotype panel were detected by both of our routine HEV assays, but as expected not by the new in‐house ratHEV assay (Table 3). No cross reactions were observed with the tested samples of the exclusivity set (*n* = 41), which contained clinical virus samples or bacterial isolates (data not shown).

### 3.7 Pilot RatHEV Surveillance

Our surveillance study included 1999 clinical plasma samples from 1773 patients, of which 19 patients were HEV (*Paslahepevirus balayani*) RNA positive (1.1%), 93 out of 197 measured patients were anti‐HEV IgG positive and 134 out of 185 measured patients were anti‐HEV IgM positive. 737 patients were immunocompromised. RatHEV RNA was not detected in any of the samples from the pilot ratHEV surveillance cohort by the assay (data not shown).

## 4 Discussion

With an increasing number of documented human cases found in case series and retrospective studies worldwide, ratHEV is considered an emerging zoonotic virus and a better understanding of prevalence, routes of infection and disease outcomes is urgently needed [11, 31]. Due to the genetic distance, it is not surprising that classical RT‐qPCR assays designed to detect *Paslahepevirus balayani* HEV genotype 1−4 RNA at the ORF2 region fail to detect ratHEV RNA due to multiple mismatches in the primer and probe sequences [16, 32, 33, 34]. We confirmed in this study that our currently used routine diagnostic RT‐qPCR assays for the detection of HEV RNA (in‐house test and commercial assay for blood screening) miss infection with ratHEV even in the case of highly positive samples. To fill the diagnostic gap, the aim of the study was to establish and validate an in‐house ratHEV RT‐qPCR assay in accordance with the European Union In Vitro Diagnostics Regulation (2017/746 EU IVDR), using the open channel of a sample to result qPCR system (cobas 5800/6800/8800). The systems feature IVD‐grade reagents, built‐in internal RNA run control, full automation, minimal hands‐on time per sample and easy scalability to different testing scenarios. Our current RT‐qPCR assay targets a single, highly conserved region within the 5′ untranslated region (5′ UTR). This region was recently shown to provide optimal analytical performance in both rodent liver and human serum samples [12]. Although primers and probes were originally designed *in silico* to ensure broad inclusivity, ongoing re‐evaluation of *in‐silico* inclusivity is warranted as additional sequence data may become available. Furthermore, the expanding genomic dataset may allow additional refinement of the assay, including incorporation of a second genomic target to enhance robustness, as recommended for diagnostic assays detecting HIV‐1 RNA or SARS‐CoV‐2 RNA [35, 36]. Our novel ratHEV RT‐qPCR assay demonstrated broad inclusivity of different *Rocahepevirus ratti* C1 strains and closely related viruses found in ferrets, mink (*Rocahepevirus ratti C2*), but not *Rocahepevirus* species C3 (Chevrier's field mouse) or *Rocahepevirus eothenomi* C4 (voles). In this study, *Rocahepevirus ratti* RNA was also detected in 11 ratHEV pre‐tested positive liver tissue samples of Norway rats from Berlin, Germany with high viral RNA loads of up to ct 11.7 and in one human plasma sample pre‐rested ratHEV positive at the Charité Berlin [19]. In line, studies from Europe and China found *Rocahepevirus ratti* RNA at high frequencies in the liver of rats and other wild rodents with positivity rates of 12.4% (63/508 rats), 6.4% (73/1136 wild rodents) and 3.8% (7/186 rats) [15, 37, 38, 39]. RatHEV showed a wide range of tissue tropism (e.g. heart, spleen, lung), with the highest HEV RNA levels detected in the liver (up to 10^8^ genome equivalents, GE/ml) [38]. In recent studies, ratHEV RNA has also been frequently detected in waste water in Italy, Spain and Sweden, highlighting the need to further investigate the role of *Rocahepevirus ratti* in human hepatitis cases of unknown origin [10, 40, 41].

The analytical performance of our assay demonstrated no cross‐reactivity when measuring a WHO HEV RNA genotype panel, clinical virus samples and bacterial isolates. LoD values were 98.9 dcp/ml in plasma and 60.3 dcp/ml in stool suspension samples, and the assay showed excellent linearity over at least 5 log steps (r2: 0.991 in plasma and 0.9989 in stool).

In this study, we retrospectively analysed 1,999 anonymized clinical plasma samples (from 1773 different patients) that were initially screened for HEV RNA. In total, 1.1% of the patients were *Paslahepevirus balayani* RNA positive (19/1773), but no ratHEV RNA positive sample was detected. In our pilot surveillance cohort no ratHEV RNA was found, implicating that ratHEV infection is a rare event and significantly less common than *Paslahepevirus balayani* infection. This finding is consistent with cohort studies from Germany (no positives in 200 samples and one positive in 2973 plasma and serum samples), Hungary (no positives in 1439 samples) and France (no positives in 224 samples) [19, 42, 43, 44]. In contrast, studies from Spain and China reported clearly higher prevalence rates of 0.24% (7/2860 samples), 15.1% (8/53 samples), 0.1% (1/842 samples) and 1.4% (8/562 samples) in their different patient cohorts [11, 12, 15, 17]. Most ratHEV infections were reported in immunocompromised or elderly patients and the clinical outcome was milder compared to HEV (*Paslahepevirus balayani*) [11, 15].

While transmission routes of ratHEV are still unclear, more clinical studies in different countries and settings are needed to define the clinical significance of ratHEV infection in different clinical settings and before routine testing strategies can be employed.

In conclusion, we adapted and validated a molecular lab‐developed RT‐qPCR assay compliant with the European Union In Vitro Diagnostics Regulation (2017/746 EU IVDR) for the detection of ratHEV RNA on a high‐throughput, easily scalable, fully automated platform. The assay is applicable to human EDTA‐plasma and stool samples in routine diagnostic settings and may contribute to a better diagnostic and understanding of ratHEV infection in humans.

## Author Contributions

Marc Lütgehetmann and Reimar Johne conceptualized and supervised the study. Jessica Panajotov, Dominik Nörz, Katja Giersch, Lisa Sophie Pflüger, Hui Ting Tang and Moritz Grunwald performed the experiments and data analysis. Marco Kaiser adapted primer/probes. Jessica Panajotov, Katja Giersch and Marc Lütgehetmann wrote the manuscript. Marco Kaiser, Susanne Pfefferle, Sven Pischke, Martin Aepfelbacher, Rainer G. Ulrich, Victor Max Corman and Reimar Johne discussed the data and corrected the manuscript. All authors agreed to the publication of the final manuscript.

## Ethics Statement

The use of patient material was conducted in accordance with §12 of the Hamburg hospital law (§12 HmbKHG). The use of anonymized remnant diagnostic samples from patients was approved and informed consent was waived by the ethics committee of the Hamburg Medical Association (PV5626).

## Conflicts of Interest

M.L. received speaker honoraria and related travel expenses from Roche Diagnostics and personal fees for participation on an advisory board from Roche Molecular Systems. D.N. received speaker honoraria and related travel expenses from Roche Diagnostics. L.S.P. received speaker honoraria from Roche Diagnostics. TIB‐MOLBIOL Syntheselabor GmbH is part of Roche group. The other authors declare no conflicts of interest.

## Supporting information


**Supporting Table 1:** Manufacturing protocol of the ratHEV RT‐qPCR assay (A) and the in‐house pan HEV RT‐qPCR assay (B) The quantities refer to one Cobas omni utility channel reagent kit cassette (Roche) with a total volume of 10.6 ml.

## Data Availability

The data that support the findings of this study are available from the corresponding author upon reasonable request.

## References

[1] B. Wang and X.‐J. Meng , “Hepatitis E Virus: Host Tropism and Zoonotic Infection,” Current Opinion in Microbiology 59 (2021): 8–15, 10.1016/j.mib.2020.07.004. 32810801 PMC7854786

[2] A. Letafati , Z. Taghiabadi , M. Roushanzamir , et al., “From Discovery to Treatment: Tracing the Path of Hepatitis E Virus,” Virology Journal 21, no. 1 (2024): 194, 10.1186/s12985-024-02470-3. 39180020 PMC11342613

[3] R. Johne , A. Plenge‐Bonig , M. Hess , R. G. Ulrich , J. Reetz , and A. Schielke , “Detection of a Novel Hepatitis E‐Like Virus in Faeces of Wild Rats Using a Nested Broad‐Spectrum RT‐PCR,” Journal of General Virology 91, no. Pt 3 (2010): 750–758, 10.1099/vir.0.016584-0. 19889929

[4] M. A. Purdy , J. F. Drexler , X.‐J. Meng , et al., “ICTV Virus Taxonomy Profile: Hepeviridae 2022,” Journal of General Virology 103, no. 9 (2022): 001778, 10.1099/jgv.0.001778. 36170152 PMC12642825

[5] G. S. Gherlan , “Rocahepevirus Ratti: An Underrecognised Cause of Acute Hepatitis,” World Journal of Hepatology 16, no. 8 (2024): 1084–1090, 10.4254/wjh.v16.i8.1084. 39221102 PMC11362906

[6] B. Wang , W. Li , J.‐H. Zhou , et al., “Chevrier's Field Mouse (Apodemus chevrieri) and Père David's Vole (Eothenomys melanogaster) in China Carry Orthohepeviruses That Form Two Putative Novel Genotypes Within the Species Orthohepevirus C,” Virologica Sinica 33, no. 1 (2018): 44–58, 10.1007/s12250-018-0011-8. 29500690 PMC6178085

[7] R. Ryll , G. Heckel , V. M. Corman , J. F. Drexler , and R. G. Ulrich , “Genomic and Spatial Variability of a European Common Vole Hepevirus,” Archives of Virology 164, no. 11 (2019): 2671–2682, 10.1007/s00705-019-04347-1. 31399875

[8] H. Bai , W. Li , D. Guan , et al., “Characterization of a Novel Rat Hepatitis E Virus Isolated From an Asian Musk Shrew (Suncus murinus),” Viruses 12, no. 7 (2020): 715, 10.3390/v12070715. 32630296 PMC7411586

[9] J. Caballero‐Gómez , S. Pereira , I. Rivero‐Calle , et al., “Acute Hepatitis in Children Due to Rat Hepatitis E Virus,” Journal of Pediatrics 273 (2024): 114125, 10.1016/j.jpeds.2024.114125. 38815747

[10] M. Casares‐Jimenez , T. Garcia‐Garcia , J. M. Suárez‐Cárdenas , et al., “Correlation of Hepatitis E and Rat Hepatitis E Viruses Urban Wastewater Monitoring and Clinical Cases,” Science of the Total Environment 908 (2024): 168203, 10.1016/j.scitotenv.2023.168203. 37914110

[11] M. Casares‐Jimenez , A. Rivero‐Juarez , P. Lopez‐Lopez , et al., “Rat Hepatitis E Virus (Rocahepevirus Ratti) in People Living With HIV,” Emerging Microbes & Infections 13, no. 1 (2024): 2295389, 10.1080/22221751.2023.2295389. 38095070 PMC10763910

[12] J. Caballero‐Gómez , M. Casares‐Jiménez , M. Gallo‐Marín , et al., “Rat Hepatitis E Virus as an Aetiological Agent of Acute Hepatitis of Unknown Origin,” Journal of Hepatology 83, no. 25 (2025): 662–669, 10.1016/j.jhep.2025.02.027. 40020930

[13] A. Rivero‐Juarez , M. Frias , A. B. Perez , et al., “Orthohepevirus C Infection as an Emerging Cause of Acute Hepatitis in Spain: First Report in Europe,” Journal of Hepatology 77, no. 2 (2022): 326–331, 10.1016/j.jhep.2022.01.028. 35167911

[14] C. Rodriguez , S. Marchand , A. Sessa , P. Cappy , and J.‐M. Pawlotsky , “Orthohepevirus C Hepatitis, an Underdiagnosed Disease?,” Journal of Hepatology 79, no. 1 (2023): e39–e41, 10.1016/j.jhep.2023.02.008. 36806365

[15] S. Sridhar , C. C.‐Y. Yip , S. Wu , et al., “Transmission of Rat Hepatitis E Virus Infection to Humans in Hong Kong: A Clinical and Epidemiological Analysis,” Hepatology 73, no. 1 (2021): 10–22, 10.1002/hep.31138. 31960460

[16] S. Sridhar , C. C. Y. Yip , S. Wu , et al., “Rat Hepatitis E Virus as Cause of Persistent Hepatitis After Liver Transplant,” Emerging Infectious Diseases 24, no. 12 (2018): 2241–2250, 10.3201/eid2412.180937. 30457530 PMC6256372

[17] S. Sridhar , C. C. Y. Yip , K. H. Y. Lo , et al., “Hepatitis E Virus Species C Infection in Humans, Hong Kong,” Clinical Infectious Diseases 75, no. 2 (2022): 288–296, 10.1093/cid/ciab919. 34718428

[18] Z. Chen , L. Wang , Y. Zhang , et al., “Substantial Spillover Burden of Rat Hepatitis E Virus in Humans,” Nature Communications 16, no. 1 (2025): 4038, 10.1038/s41467-025-59345-6. PMC12041280 40301345

[19] L. J. Mueller , M. L. Schmidt , S. Ciesek , et al., “Presence but Low Detection Rate of Rat Hepatitis E Virus in Patients in Germany 2022‐2024,” Journal of Hepatology 83, no. 3 (2025): e146–e148, 10.1016/j.jhep.2025.05.023. 40482720

[20] X. Cui , J. Situ , T. Tang , et al., “Prevalence of Rocahepevirus Ratti (Rat Hepatitis E Virus) in Humans and Rats in China,” JHEP Reports 7, no. 5 (2025): 101370, 10.1016/j.jhepr.2025.101370. 40342633 PMC12060442

[21] R. Johne , G. Heckel , A. Plenge‐Bönig , et al., “Novel Hepatitis E Virus Genotype in Norway Rats, Germany,” Emerging Infectious Diseases 16, no. 9 (2010): 1452–1455, 10.3201/eid1609.100444. 20735931 PMC3294985

[22] null Mulyanto , S. N. Depamede , M. Sriasih , et al., “Frequent Detection and Characterization of Hepatitis E Virus Variants in Wild Rats (Rattus rattus) in Indonesia,” Archives of Virology 158, no. 1 (2013): 87–96, 10.1007/s00705-012-1462-0. 22983110

[23] H. Guo , J. Xu , J. Situ , et al., “Cell Binding Tropism of Rat Hepatitis E Virus is a Pivotal Determinant of Its Zoonotic Transmission to Humans,” Proceedings of the National Academy of Sciences 121, no. 45 (2024): e2416255121, 10.1073/pnas.2416255121. PMC11551445 39467126

[24] J. Prpić and M. Baymakova , “Hepatitis E Virus (HEV) Infection Among Humans and Animals: Epidemiology, Clinical Characteristics, Treatment, and Prevention,” Pathogens 12, no. 7 (2023): 931, 10.3390/pathogens12070931. 37513778 PMC10383665

[25] S. Sridhar , J. Situ , J.‐P. Cai , et al., “Multimodal Investigation of Rat Hepatitis E Virus Antigenicity: Implications for Infection, Diagnostics, and Vaccine Efficacy,” Journal of Hepatology 74, no. 6 (2021): 1315–1324, 10.1016/j.jhep.2020.12.028. 33845058

[26] H. T. Tang , D. Nörz , M. Grunwald , et al., “Analytical and Clinical Validation of a Novel, Laboratory‐Developed, Modular Multiplex‐PCR Panel for Fully Automated High‐Throughput Detection of 16 Respiratory Viruses,” Journal of Clinical Virology 173 (2024): 105693, 10.1016/j.jcv.2024.105693. 38820916

[27] K. Giersch , D. Nörz , M. Grunwald , et al., “Adaptation and Validation of a Gastrointestinal Panel to Detect Diarrheal Virus Pathogens on a High‐Throughput qPCR System,” Medical Microbiology and Immunology 214, no. 1 (2025): 28, 10.1007/s00430-025-00837-z. 40459771 PMC12134015

[28] J. Panajotov , A. Falkenhagen , A. K. Gadicherla , and R. Johne , “Molecularly Generated Rat Hepatitis E Virus Strains From Human and Rat Show Efficient Replication in a Human Hepatoma Cell Line,” Virus Research 344 (2024): 199364, 10.1016/j.virusres.2024.199364. 38522562 PMC10995862

[29] J. Panajotov , K. Schilling‐Loeffler , C. Mehl , et al., “Long‐Term Circulation and Molecular Evolution of Rat Hepatitis E Virus in Wild Norway Rat Populations From Berlin, Germany,” Infection, Genetics and Evolution 135 (2025): 105841, 10.1016/j.meegid.2025.105841. 41109490

[30] J. A. Garson , R. B. Ferns , P. R. Grant , et al., “Minor Groove Binder Modification of Widely Used TaqMan Probe for Hepatitis E Virus Reduces Risk of False Negative Real‐Time PCR Results,” Journal of Virological Methods 186, no. 1–2 (2012): 157–160, 10.1016/j.jviromet.2012.07.027. 22871672

[31] S. Benavent , S. Carlos , and G. Reina , “Rocahepevirus Ratti as an Emerging Cause of Acute Hepatitis Worldwide,” Microorganisms 11, no. 12 (2023): 2996, 10.3390/microorganisms11122996. 38138140 PMC10745784

[32] P. Colson , C. Coze , P. Gallian , M. Henry , P. De Micco , and C. Tamalet , “Transfusion‐Associated Hepatitis E, France,” Emerging Infectious Diseases 13, no. 4 (2007): 648–649, 10.3201/eid1304.061387. 17561564 PMC2725983

[33] N. Jothikumar , T. L. Cromeans , B. H. Robertson , X. J. Meng , and V. R. Hill , “A Broadly Reactive One‐Step Real‐Time Rt‐PCR Assay for Rapid and Sensitive Detection of Hepatitis E Virus,” Journal of Virological Methods 131, no. 1 (2006): 65–71, 10.1016/j.jviromet.2005.07.004. 16125257

[34] K. J. Rolfe , M. D. Curran , N. Mangrolia , et al., “First Case of Genotype 4 Human Hepatitis E Virus Infection Acquired in India,” Journal of Clinical Virology 48, no. 1 (2010): 58–61, 10.1016/j.jcv.2010.02.004. 20227909

[35] K. Shah , V. Ragupathy , A. Saga , and I. Hewlett , “High Sensitivity Detection of HIV‐1 Using Two Genomic Targets Compared With Single Target PCR,” Journal of Medical Virology 88, no. 6 (2016): 1092–1097, 10.1002/jmv.24431. 26575693

[36] L. Pezzi , R. N. Charrel , L. Ninove , et al., “Development and Evaluation of a Duo SARS‐CoV‐2 RT‐qPCR Assay Combining Two Assays Approved by the World Health Organization Targeting the Envelope and the RNA‐Dependant RNA Polymerase (RdRp) Coding Regions,” Viruses 12, no. 6 (2020): 686, 10.3390/v12060686. 32630601 PMC7354606

[37] R. Ryll , S. Bernstein , E. Heuser , et al., “Detection of Rat Hepatitis E Virus in Wild Norway Rats (Rattus Norvegicus) and Black Rats (Rattus Rattus) From 11 European Countries,” Veterinary Microbiology 208 (2017): 58–68, 10.1016/j.vetmic.2017.07.001. 28888650

[38] Q. Ding , B. Hu , X. Yao , et al., “Prevalence and Molecular Characterization of Hepatitis E Virus (HEV) From Wild Rodents in Hubei Province, China,” Infection, Genetics and Evolution 121 (2024): 105602, 10.1016/j.meegid.2024.105602. 38734397

[39] W. Li , D. Guan , J. Su , et al., “High Prevalence of Rat Hepatitis E Virus in Wild Rats in China,” Veterinary Microbiology 165, no. 3–4 (2013): 275–280, 10.1016/j.vetmic.2013.03.017. 23623690

[40] M. P. Churqui , M. Ghaleb , T. Tunovic , et al., “High Prevalence of Hepatitis E and Rat Hepatitis E Viruses in Wastewater in Gothenburg, Sweden,” One Health 19 (2024): 100882, 10.1016/j.onehlt.2024.100882. 39267918 PMC11391864

[41] A. Palombieri , F. Di Profio , V. Sarchese , et al., “Surveillance for Rat Hepatitis E in Wastewater Networks, Italy,” Microbiology Spectrum 11, no. 6 (2023): e0267523, 10.1128/spectrum.02675-23. 37850788 PMC10714833

[42] P. Pankovics , O. Némethy , Á. Boros , G. Pár , P. Szakály , and G. Reuter , “Four‐Year Long (2014‐2017) Clinical and Laboratory Surveillance of Hepatitis E Virus Infections Using Combined Antibody, Molecular, Antigen and Avidity Detection Methods: Increasing Incidence and Chronic HEV Case in Hungary,” Journal of Clinical Virology 124 (2020): 104284, 10.1016/j.jcv.2020.104284. 32007844

[43] M. Faber , J. J. Wenzel , M. Erl , K. Stark , and M. Schemmerer , “No Evidence for Orthohepevirus C in Archived Human Samples in Germany, 2000‐2020,” Viruses 14, no. 4 (2022): 742, 10.3390/v14040742. 35458471 PMC9029421

[44] D. Parraud , S. Lhomme , J. M. Péron , et al., “Rat Hepatitis E Virus: Presence in Humans in South‐Western France?,” Frontiers in Medicine 8 (2021): 726363, 10.3389/fmed.2021.726363. 34540871 PMC8448288

