# Supplementary material for: Development of a Fully Automated, High‐Throughput Molecular Assay for Detection of Rat Hepatitis E Virus in Routine Diagnostics
Source: J Med Virol. 2026 Feb 9;98(2):e70824. doi: 10.1002/jmv.70824 (PMC12884570; doi:10.1002/jmv.70824)
Supplement: Supplementary file 1 — Supporting Table 1: Manufacturing protocol of the ratHEV RT‐qPCR assay (A) and the in‐house pan HEV RT‐qPCR assay (B) The quantities refer to one Cobas omni utility channel reagent kit cassette (Roche) with a total volume of 10.6 ml. [file JMV-98-e70824-s001.pdf]

Development of a fully automated, high-throughput molecular assay for detection of rat hepatitis E virus in routine diagnostics

Jessica Panajotov[1#], Katja Giersch[2#], Lisa Sophie Pflüger[2], Dominik Nörz[2], Moritz Grunwald[2], Hui Ting Tang[2], Marco Kaiser[3], Sven Pischke[4,5], Rainer G. Ulrich[5,6], Susanne Pfefferle[2], Julian Schulze zur Wisch[4,5], *Victor Max Corman*[7,8,9], Martin Aepfelbacher[2], Reimar Johne[1] and Marc Lütgehetmann[2,5,*]

1 German Federal Institute for Risk Assessment, Berlin, Germany.

2 University Medical Center Hamburg-Eppendorf (UKE), Institute of Medical Microbiology, Virology and Hygiene, Hamburg, Germany.

3 TIB-Molbiol Syntheselabor GmbH, Berlin.

4 Department of Internal Medicine, University Medical Center Hamburg-Eppendorf, Hamburg, Germany.

5 German Center for Infection Research (DZIF), Hamburg-Lübeck-Borstel-Riems site, Germany.

6 Friedrich-Loeffler-Institut, Institute of Novel and Emerging Infectious Diseases, Greifswald-Insel Riems, Germany.

7 Institute of Virology, Charité-Universitätsmedizin Berlin, corporate member of Freie Universität Berlin and Humboldt-Universität zu Berlin, Berlin, Germany.

8 German Centre for Infection Research (DZIF), associated partner site Charité, Berlin, Germany.

9 Labor Berlin-Charité Vivantes, Berlin, Germany.

# shared authorship

* corresponding author

Correspondence to:

Dr. Marc Lütgehetmann

Institute of Medical Microbiology, Virology and Hygiene, University Medical Center Hamburg-Eppendorf

Martinistraße 52, D-20246 Hamburg

mluetgeh@uke.de

SUPPLEMENTARY METHODS

The cobas omni Utility Channel Reagent Kit (Roche) comprises the protease, elution buffer, Master Mix Reagent 1 (MMx-R1) as well as the internal RNA full-process control (IC), which is provided as a packaged RNA spike within the assay cassette and which is an integral part of the reagent system. IC target-specific primers and probes are already included in the Master Mix Reagent 2 (MMx-R2), which also contains a polymerase with reverse transcriptase activity required for amplification of RNA targets. Detection of the internal RNA control is performed exclusively in channel 5 (excitation 680 ± 10 nm, emission 730 ± 30 nm), a channel dedicated solely to for this purpose and to avoid interference with target detection.

Target primer stock solutions (500 µM) and probe stock solutions (100 µM) were added to 10 ml of MMx-R2 in a light-protected tube and carefully mixed by inverting the tube 20 times, resulting in a total volume of 10.6 ml. The manufacturing protocol of the ratHEV RT-qPCR assay and the in-house pan HEV RT-qPCR assay are shown in **Supplementary Table 1**. The prepared master mix with target primers and probes was then pipetted in the reagent cassette according to the manufacturer´s instructions. The cassette should be stored at 2–8 °C until use, for a maximum of six months. However, each laboratory needs to evaluate the stability of the cassette for their own conditions.

A

components	stock concentration (in µM)	1 cassette (in µl)
MMX-R2	-	10000.00
H2O	-	457.80
HEC-qual-FO1	500	28.00
HEC-qual-FO2	500	28.00
HEC-qual-RO	500	58.70
HEC-probe	100	27.50

B

components	stock concentration (in µM)	1 cassette (in µl)
MMX-R2	-	10000.00
H2O	-	493.7
HEV_FO	500	29.3
HEV_R	500	58.7
HEV_HEX	100	18.30

Supplementary Table 1. *Manufacturing protocol of the ratHEV RT-qPCR assay (**A**) and the in-house pan HEV RT-qPCR assay (**B**). The quantities refer to one Cobas omni utility channel reagent kit cassette (Roche) with a total volume of 10.6 ml.*
